# Effects of mechanical ventilation on the interstitial extracellular matrix in healthy lungs and lungs affected by acute respiratory distress syndrome: a narrative review

**DOI:** 10.1186/s13054-024-04942-y

**Published:** 2024-05-15

**Authors:** Lou’i Al-Husinat, Saif Azzam, Sarah Al Sharie, Ahmed H. Al Sharie, Denise Battaglini, Chiara Robba, John J. Marini, Lauren T. Thornton, Fernanda F. Cruz, Pedro L. Silva, Patricia R. M. Rocco

**Affiliations:** 1 Department of Clinical Sciences, Faculty of Medicine, Yarmouk University, Irbid, Jordan; 2 Faculty of Medicine, Yarmouk University, Irbid, Jordan; 3 Department of Pathology and Microbiology, Jordan University of Science and Technology, Irbid, Jordan; 4 Anesthesia and Intensive Care, IRCCS Ospedale Policlinico San Martino, Genoa, Italy; 5 Dipartimento di Scienze Chirurgiche e Diagnostiche, Università Degli Studi di Genova, Genoa, Italy; 6 Department of Pulmonary and Critical Care Medicine, University of Minnesota, Minneapolis, St Paul, MN USA; 7 Laboratory of Pulmonary Investigation, Institute of Biophysics Carlos Chagas Filho, Federal University of Rio de Janeiro, Rio de Janeiro, Brazil

**Keywords:** Ventilator-induced lung injury, Extracellular matrix, Mechanical ventilation, Collagen fiber, Acute respiratory distress syndrome

## Abstract

**Background:**

Mechanical ventilation, a lifesaving intervention in critical care, can lead to damage in the extracellular matrix (ECM), triggering inflammation and ventilator-induced lung injury (VILI), particularly in conditions such as acute respiratory distress syndrome (ARDS). This review discusses the detailed structure of the ECM in healthy and ARDS-affected lungs under mechanical ventilation, aiming to bridge the gap between experimental insights and clinical practice by offering a thorough understanding of lung ECM organization and the dynamics of its alteration during mechanical ventilation.

**Main text:**

Focusing on the clinical implications, we explore the potential of precise interventions targeting the ECM and cellular signaling pathways to mitigate lung damage, reduce inflammation, and ultimately improve outcomes for critically ill patients. By analyzing a range of experimental studies and clinical papers, particular attention is paid to the roles of matrix metalloproteinases (MMPs), integrins, and other molecules in ECM damage and VILI. This synthesis not only sheds light on the structural changes induced by mechanical stress but also underscores the importance of cellular responses such as inflammation, fibrosis, and excessive activation of MMPs.

**Conclusions:**

This review emphasizes the significance of mechanical cues transduced by integrins and their impact on cellular behavior during ventilation, offering insights into the complex interactions between mechanical ventilation, ECM damage, and cellular signaling. By understanding these mechanisms, healthcare professionals in critical care can anticipate the consequences of mechanical ventilation and use targeted strategies to prevent or minimize ECM damage, ultimately leading to better patient management and outcomes in critical care settings.

## Key points

Question: How does mechanical ventilation affect the lung’s interstitial extracellular matrix (ECM), with a specific focus on ECM organization in healthy lungs and those affected by acute respiratory distress syndrome (ARDS).

Findings: The narrative review reveals that mechanical ventilation induces stress on the interstitial ECM, leading to inflammation, fibrogenesis, elastogenesis, and matrix metalloproteinase hyperactivity. Matrix metalloproteinase and integrin involvement in ECM damage are emphasized. Our findings are based on a narrative review of studies of mechanical ventilation-induced alterations of interstitial ECM organization and cellular response.

Meaning: The key conclusion is that understanding the intricate processes of interstitial ECM damage, as well as cell signaling and communication related to ventilation-induced lung injury, can pave the way for more targeted interventions that have the potential to mitigate lung damage and improve outcomes for patients in critical care settings.

## Background

The extracellular matrix (ECM) is an intricate structure composed of proteins, polysaccharides, and various other components that envelop and give structure to cells [1]. The primary elements of the ECM include collagen, elastin, proteoglycans, glycosaminoglycans (GAGs), and fibrinogen, which establish the fundamental architecture of bodily tissues. These constituents offer mechanical strength and flexibility, helping to maintain normal fluid dynamics within tissues, providing an efficient low-resistance channel for gas-exchange, regulating cellular activities through the binding of growth factors, chemokines, cytokines, and interactions with cell-surface receptors, and facilitating tissue repair and remodeling [1].

Mechanical ventilation, although lifesaving, can induce excessive stress and strain due to alveolar collapse and overdistension, leading to inflammation, fibrogenesis, elastogenesis, and matrix metalloproteinase hyperactivity, followed by ventilator-induced lung injury (VILI) [2]. VILI has potential to exacerbate respiratory distress, delay healing, and compromise patient outcomes [3]. As the complex interplay of physiologic processes unfolds within the intricate landscape of the respiratory system, the role of the ECM emerges as a crucial determinant in the genesis of VILI [2]. Thus, the influence of the ECM extends beyond its conventional supportive role, extending to mechanotransduction and inflammatory modulation [4]. Under high mechanical stress, ECM components change, which, in turn, affects the delicate balance between tissue stiffness and flexibility, potentiating the propagation of VILI [5, 6]. Understanding of the mechanical stress imparted by mechanical ventilation is essential to comprehend the involvement of the interstitial ECM in the pathophysiology of VILI [4, 7].

Cellular signaling amplifies the significance of the ECM in the genesis of VILI. Integrins, transmembrane receptors that facilitate cell–ECM adhesion, act as intermediaries in mechanochemical communication [8]. In response to mechanical cues generated during mechanical ventilation, integrins transduce signals that regulate processes ranging from cell survival to inflammation [9]. The composition and spatial organization of the ECM influence integrin-mediated signaling, thereby contributing to the intricate orchestration of inflammatory responses and tissue repair [10].

The interstitial ECM, which is the focus of this review, is found within the spaces surrounding lung cells and fibroblasts, encompassing a network of fibers (e.g., collagen and elastic fibers) [11]. This matrix is crucial for maintaining the structural integrity of the lung, ensuring appropriate mechanical properties for gas-exchange, and mediating cellular responses to mechanical stress [12]. In contrast, the intraluminal ECM, located within the luminal spaces of the lung, including airways and alveoli, primarily interacts with the air we breathe and can be influenced by factors such as inhaled particles and pathogens [13, 14]. Mechanical ventilation imposes mechanical stress that predominantly affects the interstitial ECM, leading to alterations in its composition and organization [15]. While several studies have investigated ECM alterations in the context of mechanical ventilation, distinguishing between changes in the interstitial and intraluminal compartments remains challenging [16]. Most biomarker studies, for example, cannot differentiate between shedding or remodeling of ECM components originating from these distinct compartments [17].

We present a comprehensive review of the interstitial ECM organization in the lungs, mechanisms of mechanotransduction, and ECM modifications during mechanical ventilation in healthy lungs and lungs affected by acute respiratory distress syndrome (ARDS).

## Extracellular matrix organization

Lung interstitial ECM consists of basement membranes beneath the cellular epithelium and endothelium, and interstitial spaces containing fibroblasts [11]. The ECM interacts with cells to guide their development and serves multiple functions to preserve cellular homeostasis, including support, tissue segregation, receptor regulation, and pH stabilization [18, 19].

**Fig. 1:**
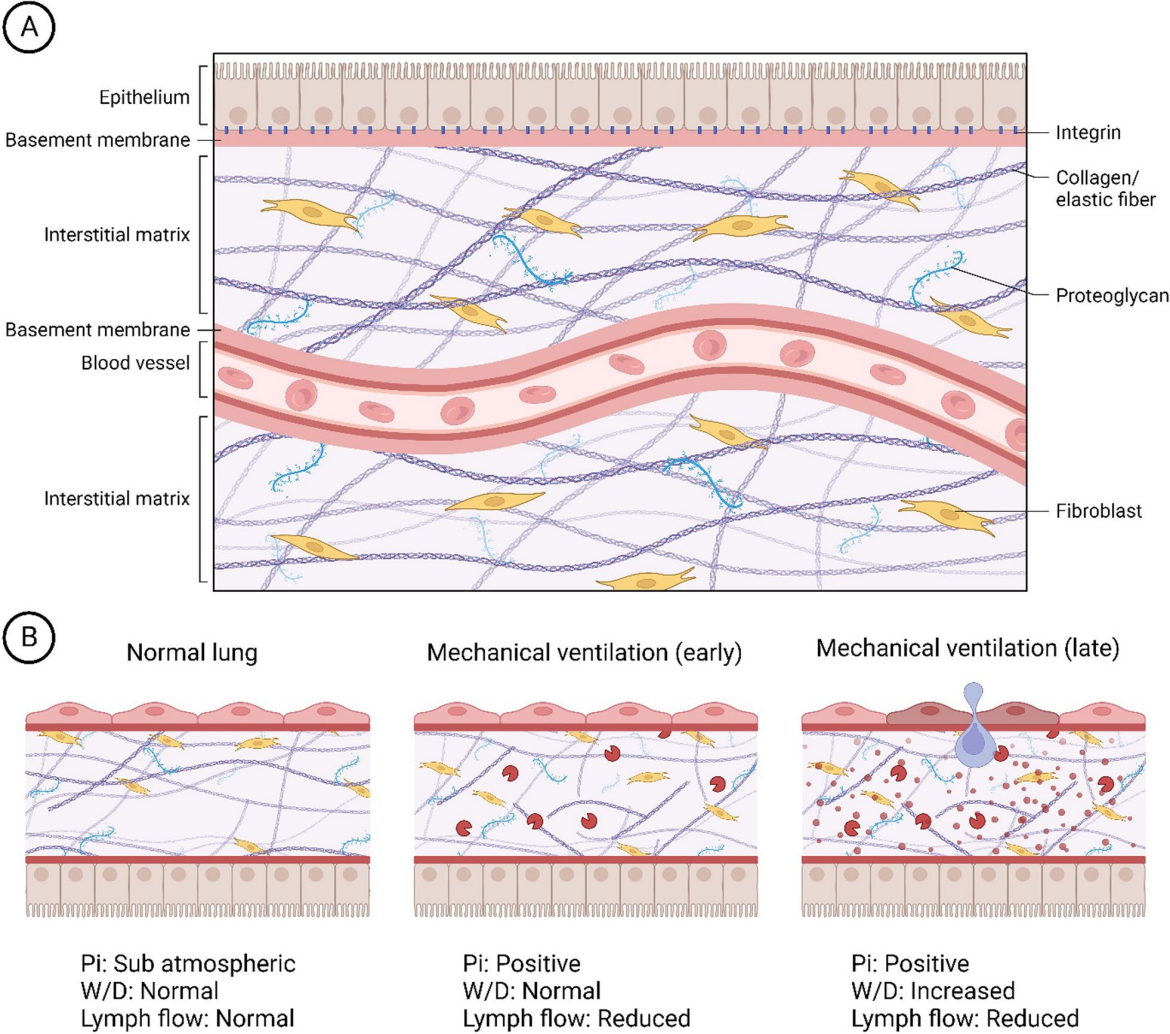
**A** Normal organization of the extracellular matrix. **B** Changes in the extracellular matrix occur during mechanical ventilation in both early and late phases. Key indicators include interstitial pressure (Pi), the wet-to-dry ratio (W/D), chondroitin sulfate, heparan sulfate, and proteoglycan. Matrix metalloproteinases (MMPs) also play a role. In the initial stages of mechanical ventilation, there is a predominant occurrence of glycosaminoglycan shedding and proteoglycan fragmentation, initiating the inflammatory process and activating MMPs. Subsequently, damage to the alveolar capillary membrane, collagen deposition, and the development of alveolar and interstitial edema occur, ultimately leading to acute respiratory distress syndrome. Figure generated by BioRender

The ECM is composed of macromolecules (Fig. 1): 1. Glycoproteins, including fibronectin, play a crucial role in wound healing, clot formation and ECM assembly [19–22]; 2. GAGs, including sulfated GAGs (heparan sulfate, chondroitin sulfate, dermatan sulfate, and keratan sulfate) [23], are expressed on the cell surface [24] and interact with many proteins [e.g., interleukin (IL)-8 [25], and lipid-binding proteins [26]]. Hyaluronic acid, the most abundant and the largest non-sulfated GAG, is involved in tissue repair, hydration, and protection against infections [27]; 3. Proteoglycans (PGs), including chondroitin sulfate-containing proteoglycans (CS-PG; versican), heparan sulfate proteoglycans (HS-PG; perlecan and glypican), chondroitin and heparan sulfate-containing PGs (CS-HS-PGs; syndecan), and dermatan sulfate-containing PGs (DS-PG; decorin) [28], contain at least one GAG in their structure, which shares a covalent bond with a core protein [29]. Versican is localized in the lung interstitium, perlecan in the vascular basement membrane, decorin in the interstitium and epithelial basement membrane, and syndecan and glypican in the cell surface. They serve several functions during wound healing and help modulate lung inflammation and remodeling [30]; and 4. Fibrous proteins include fibrillar (types I, II, and III) and non-fibrillar or amorphous collagens (types IV, V, VI) [25, 26]. Elastic fibers provide elasticity and are abundant in the sub epithelium of alveolar septa [31].

The ECM undergoes dynamic processes of synthesis and breakdown influenced by multiple proteins and enzymes. In this line, matrix metalloproteinases (MMPs) are the most prominent and can lead to GAG shedding, thus causing lung injury [1]. MMP proteolytic activity is tightly regulated by the tissue inhibitors of metalloproteinases (TIMPs) [32, 33]. The distinction between MMP activity in the interstitial versus intraluminal ECM is not well-defined [34].

## Mechanotransduction mechanisms

**Fig. 2:**
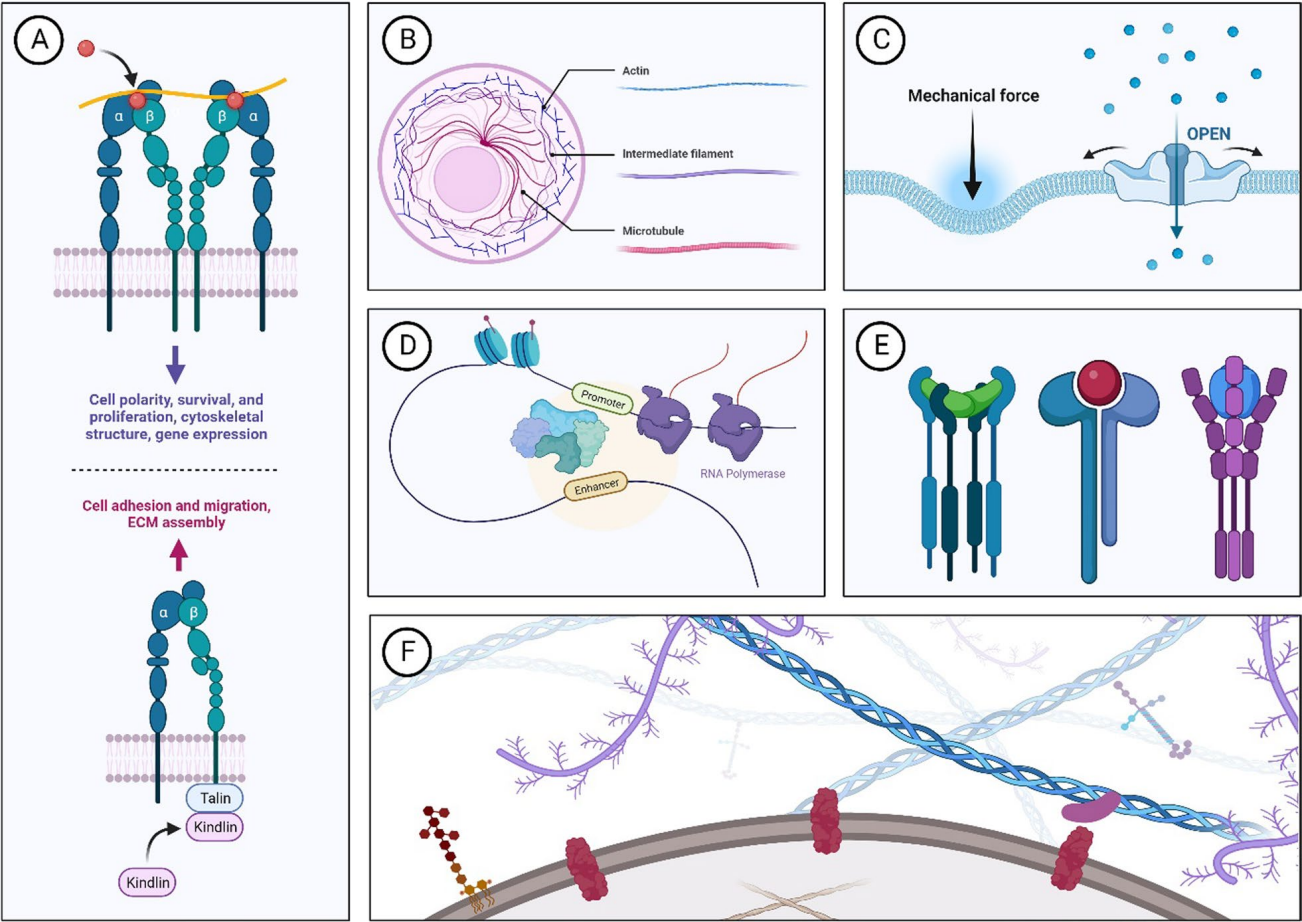
Key mechanisms involved in mechanotransduction within the extracellular matrix (ECM) include: **A** integrins and focal adhesions; **B** cytoskeletal tension; **C** mechanosensitive ion channels; **D** nuclear deformation and gene expression; **E** soluble factors and paracrine signaling; **F** extracellular matrix remodeling and matrix stiffness and topography. Figure generated by BioRender

Mechanotransduction refers to the process by which cells convert mechanical stimuli from their environment into biochemical signals [35]. Figure 2 shows the key mechanisms involved in mechanotransduction within the ECM including: 1) Integrins and focal adhesions. When mechanical stress is applied, integrins cluster together and recruit a variety of intracellular proteins to form focal adhesions [36, 37]; 2) Cytoskeletal tension. The cytoskeleton, a network of fibers within the cell that maintains shape and facilitates movement, consists mainly of actin filaments, microtubules, and intermediate filaments [38]. Mechanical forces can cause the cytoskeleton to either stretch or contract, leading to changes in cell tension, which can activate signaling pathways that regulate cell function [39]; 3) Mechanosensitive ion channels. Mechanosensitive ion channels are a diverse group of transmembrane proteins that can open or close in response to mechanical forces [40]. They can be categorized into two groups based on their activation mechanism [41]. In direct mechanogating, the ion channel is mechanically deformed or stretched by external forces, leading to a conformational change that opens the channel pore [42]. In indirect mechanogating, the ion channel is activated by secondary messengers or cytoskeletal elements that are themselves affected by mechanical forces [43]. Several families of mechanosensitive ion channels have been identified, including the Piezo channels, a class of ion channels that are critically involved in mechanotransduction [44–46]. In the lungs, Piezo2 help detect and respond to changes in lung stretch, which is essential for maintaining proper lung function and protecting against VILI [47]. Moreover, Transient Receptor Potential (TRP) channels [48] respond to mechanical forces and changes in pH levels, broadening their role in sensory and physiologic processes [49]. The K2P (Two-Pore Domain Potassium) channels are sensitive to various physiologic stimuli, including pH changes, mechanical stretch, temperature, and various pharmacological agents [50]. The mechanosensitivity of certain K2P channels, such as TREK-1, suggests a potential link between these channels and the development of VILI [50, 51]. Furthermore, K2P channel activity could affect the secretion of inflammatory mediators, the recruitment of immune cells to the lung, and the regulation of vascular tone, all of which are relevant in the context of VILI [52]; 4) Nuclear deformation and gene expression. Mechanical forces can be transmitted to the nucleus, leading to changes in its shape and structure [53], which can influence gene expression by altering the physical organization of chromatin and the accessibility of transcription factors to DNA [54, 55]; 5) Soluble factors and paracrine signaling. Mechanical stress can lead to the release of growth factors, cytokines, and other signaling molecules that can diffuse through the ECM and influence the behavior of nearby cells [56]. 6) Extracellular matrix remodeling. Mechanical forces can remodel the ECM by secreting MMPs that degrade ECM components [57]. Remodeling can also involve the synthesis of new ECM proteins with the potential to alter its mechanical properties and affect neighboring cells [58]. 7) Matrix stiffness and topography. ECM stiffness directly influences cellular responses [59]. This stiffness varies across tissues and changes dynamically during disease development, progression, and healing [5]. Cells sense this stiffness through focal adhesions, which connect the ECM to the cell's cytoskeleton via transmembrane proteins (e.g. integrins) [36]. The ECM's topographic features (geometry, orientation, and texture) influence cell alignment, shape, and function [60]. The surface roughness and patterning of the ECM can also affect cell motility, sensing capabilities, and the development of cellular structures [61]. The translation of mechanical cues from ECM stiffness and topography into cellular actions occurs through mechanotransduction pathways [62], which modulate gene expression, cell cycle progression, and apoptosis, tailoring cellular responses to the mechanical environment [63].

## Effects of mechanical ventilation on the ECM in healthy lungs

The main mechanisms of VILI, barotrauma/volutrauma, atelectrauma, and biotrauma may lead to ECM damage [64–68]. Low tidal volume (V_T_) may lead to damage to the peripheral airways and interstitium via atelectrauma, whereas positive end-expiratory pressure (PEEP) may negate such alterations [65]. Higher V_T_ may also induce lung damage [66], with injury attributable to increased transpulmonary pressure, maldistribution of intrathoracic pressures, alterations of vascular flows, and reduction of lymphatic drainage of the lung. The latter may increase interstitial fluid volume, and abnormally distributed pressure may increase mechanical stress on the ECM. High V_T_ increases the expression of versican, HS-PG, and biglycan in lung tissue, thereby stimulating inflammation [67, 68].

Higher peak airway pressures increase type III procollagen (PCIII) mRNA expression (94). PCIII, the earliest collagen to undergo remodeling during lung fibrogenesis, is non-specific for lung injury, but has been utilized as a preliminary indicator of lung parenchyma remodeling [69].

Although the hydrostatic pressure of the interstitium (Pi) is negative in healthy lungs, Pi increases in mild edema, and approaches zero during severe edema. The negative Pi of the normal state regulates and maintains fluid filtration into the interstitium. In the mild edematous state, the Pi increases significantly with only a small increase in interstitial fluid volume, an attenuation which can be explained by low tissue compliance. This “stiffness” of the fibrous components of the ECM halts the movement of fluid into the interstitial space, impeding progression from mild to severe pulmonary edema [1, 69].

Mechanical ventilation leads to damage of GAGs and PGs in the pulmonary ECM, including HS-PG and CS-PG. Fragmentation of HS-PG and CS-PG results in pulmonary edema [70–73].

MMP-2 is effective mainly in the degradation of type IV collagen and other basement membrane components. Most MMPs are not active in normal healthy tissues, but are expressed in diseased tissues that are inflamed or undergoing repair and remodeling [33]. MMP-2 and MMP-9 are increased during mechanical ventilation, depending on the amplitude of V_T_. Mechanical ventilation may enhance the degradation capabilities of MMPs, causing GAG shedding [71].

## Effects of mechanical ventilation on the ECM in lungs affected by ARDS

Despite advances in ARDS management, autopsies show pulmonary fibrosis in > 50% of patients [74]. Abnormal lung remodeling may be profoundly affected by different ventilator settings such as V_T_, PEEP, airway and transpulmonary pressures, and respiratory rate, which can promote interstitial ECM damage in ARDS [75]. A summary of ECM changes associated with VILI is presented in Fig. 1B.

### VT and PEEP

Mechanical ventilation using high V_T_ has been associated with damage to GAGs, which may lead to increased lung edema [76–78] and inflammation [79], the latter through the interaction of GAGs with chemokines (e.g. IL-8) [80]. Additionally, high V_T_ can induce lung fibrosis through secretion of extracellular vesicles, and this might be associated with activation of the c-Jun N-terminal kinase (JNK) signaling pathway [81]. Inhibition of the JNK pathway may reduce the severity of fibrosis in vivo. Therefore, inhibition of extracellular vesicle secretion and activation of the JNK signaling pathway are promising strategies for treating pulmonary fibrosis induced by mechanical ventilation.

The actual value of V_T_ relative to inflation capacity and different patterns of increasing V_T_ may affect the ECM in experimental ARDS [82]. An abrupt increase to high V_T_ leads to heightened expression of MMP-9 and syndecan, which may contribute to lung inflammation. MMPs are secreted by fibroblasts via the action of extracellular matrix metalloproteinase inducer [83, 84] and the MMP inhibitor prinomastat decreases lung injury [85]. Some fragmented ECM proteins, called matrikines (elastin, osteopontin, and versican), can act as ligands to interact with certain receptors [86], leading to inflammation [87].

Even at low mechanical power [88], mechanical ventilation with high V_T_ also affects the ECM by increasing MMP-9 and syndecan. This observation is in contrast with the hypothesis that so long as mechanical power is kept below a safe threshold, high V_T_ should not be injurious. Therefore, to mitigate VILI, controlling V_T_ seems to determine the importance of controlling respiratory rate.

High PEEP levels may lead to increased stress in lung parenchyma, ECM, and lung vessels. At lower V_T_, PEEP appears protective against VILI, preventing GAG shedding [89], whereas, higher PEEP increased the expression of procollagen, fibronectin, transforming growth factor (TGF)-β1, and basic fibroblast growth factor [90].

Mechanical stress can damage the ECM by affecting gene expression of the connective tissue growth factor (CTGF) [91], thus increasing TGF-β and promoting the synthesis of collagens I and III [92–94]. TGF-β can potentially contribute to alveolar damage and the development of pulmonary edema because it hinders lung fluid clearance [95, 96].

### Recruitment maneuvers

The influence of RMs on ECM injury response to ventilation has been explored experimentally [97–99]. In this line, RMs (40 cmH_2_O continuous positive airway pressure for 40 s) resulted in differing ECM damage when applied in lungs with ARDS [97]. In models of pulmonary and extrapulmonary ARDS with similar mechanical compromise [98], RMs increase PCIII expression only in pulmonary ARDS. The effects of abrupt, gradual, and more gradual increments in PEEP [99] were also studied in experimental ARDS. Both gradual increment groups were associated with a decrease in gene expression of syndecan and decorin, emphasizing the relative benefit of gradual PEEP increments for patients with ARDS.

### Transpulmonary pressure

Transpulmonary pressure represents the stress imposed locally on the lung parenchyma and, therefore, the risk of VILI. Increasing global transpulmonary pressure from 5 to 20 cmH_2_O has been associated with ~ tenfold greater number of endothelial and epithelial breaks [100]. The effects of pressure and volume on lung tissue strips were evaluated using oscillation to induce stress. An increase in PCIII expression was found with stress induced by pressure (representing force) but not by volume (representing amplitude) [78]. The relationship between PCIII and force was linear (with amplitude held constant). That study suggests the existence of a *threshold* level of stress, whereby increasing the volume (amplitude) modestly may not increase stress to the threshold level, whereas increasing the amplitude further might do so. Crossing the threshold would then stimulate fibroblasts.

Different combinations of V_T_ and PEEP generate different transpulmonary driving pressures in experimental ARDS. In one comparative study, the combination of low V_T_ and PEEP (low transpulmonary driving pressure) resulted in the least PCIII expression, regardless of the presence of alveolar collapse [101].

### Different modes of mechanical ventilation

In experimental ARDS, fibroblasts are activated according to the mode of mechanical ventilation. Time-controlled adaptive ventilation, compared with volume-controlled ventilation, led to less ECM damage due to better distribution of transpulmonary pressure [104]. With assisted mechanical ventilation, Neurally Adjusted Ventilatory Assist (NAVA), variable pressure support ventilation (Noisy PSV), and conventional pressure-controlled ventilation (PCV) resulted in similar lung injuries and incidence of asynchrony [102]. Noisy PSV led to lower PCIII expression than NAVA and PCV, which may be associated with different lung distribution of stress and strain. Regardless of the mode of ventilation (PCV *versus* PSV), sigh decreased PCIII expression in pulmonary ARDS but increased PCIII in extrapulmonary ARDS [103], suggesting the importance of ARDS etiology on the susceptibility of ECM to damage [104].

In short, changes in V_T_, PEEP, recruitment maneuver, transpulmonary pressure, and modes of mechanical ventilation affect the ECM differently.

### ECM damage and inflammation

Mechanical ventilation with high peak inspiratory pressure elicits early signs of inflammation, including changes in the ECM [105]. GAGs and PGs regulate inflammatory responses in the lungs. Endocan is increased in ARDS patients and is thought to be a useful marker to indicate the progression of organ dysfunction [106]. GAG and PG fragments can activate toll-like receptors [107], promote leukocyte adhesion and migration, as well as activate MMP-2 [108].

## Clinical implications and future perspectives

Identifying early markers of ECM damage and lung injury holds promise for implementation of preventive strategies against ARDS, particularly in patients supported by mechanical ventilation [16]. Early detection facilitates timely interventions to mitigate lung injury, thereby improving outcomes [109]. Timely intervention and risk stratification are paramount to improving respiratory management [110]. Key potential biomarkers include urinary desmosine levels, indicative of ECM degradation and lung damage; surfactant proteins (SPs) reflecting alveolar-capillary barrier integrity; inflammatory cytokines such as IL-6 and IL-8, highlighting inflammation; and plasma biomarkers such as angiopoietin-2 and receptor for advanced glycation end-products (RAGE), associated with endothelial and epithelial cell damage, respectively [111]. These biomarkers could revolutionize the management of patients at risk for ARDS by enabling personalized, lung-protective strategies and dynamic care adjustments [112]. However, the clinical application of these biomarkers requires further validation to confirm their predictive value and cost-effectiveness [113].

McClintock et al. [114] found that ARDS patients who died had a higher desmosine-to-creatinine ratio than ARDS patients who survived. They found that the increase in urine desmosine levels in patients ventilated with lower V_T_ was attenuated, meaning less elastin degradation, and the mortality rate was lower. Tenholder et al. [115] found that excreted desmosine levels were significantly higher in ARDS patients than in patients with cardiogenic pulmonary edema and non-pulmonary edema. These findings suggest that simple laboratory tests eventually might be used to identify the presence of elastin degeneration products, such as desmosine, thereby predicting the onset of ARDS or iatrogenic lung injury in mechanically ventilated patients.

Optimization of mechanical ventilation strategies, including the use of lower V_T_, PEEP, and prone positioning, appears crucial for minimizing lung injury in patients with or at risk of ARDS [116]. These interventions may be informed by understanding the dynamics of the ECM and early biomarkers of lung injury [117], guiding the adjustment of ventilation parameters to protect lung tissue. There are some pre-clinical studies that tried to use biomarkers to identify lung damage according to specific ventilatory strategies [82, 99, 118–120]. In a very elegant study, Ranieri et al. [121] reported that non-protective mechanical ventilation can induce a cytokine response that may be attenuated by a strategy to minimize overdistention and recruitment/derecruitment of the lung. There are several obstacles when evaluating biomarkers in mechanically ventilated patients with ARDS, such as: the moment of analysis (the peak of biomarkers varies according to the time course of the disease), risk factors of ARDS (e.g. diabetes, immunosuppressors), and ARDS etiology [in pulmonary versus extrapulmonary ARDS, earlier lung epithelial cell damage is observed with the predominance of specific biomarkers (e.g. SP-D, RAGE)] [122]. To mitigate obstacles presented by the heterogeneous population with ARDS, personalized mechanical ventilation strategies should be applied [122].

Pharmacological interventions targeting ECM remodeling and inflammation offer promising strategies for mitigating lung injury by preserving ECM integrity and promoting lung repair [123]. Key approaches include the use of MMP inhibitors, anti-inflammatory agents, such as corticosteroids and cytokine inhibitors (e.g. IL-6 inhibitor), and growth factors (TGF-β, vascular endothelial growth factor, and epidermal growth factor) that promote cell proliferation and repair of damaged lung tissues, which is essential for maintaining ECM integrity and restoring lung function [124].

Stern et al. [125] detected levels of keratinocyte growth factor (KGF) in bronchoalveolar lavage fluids of ARDS patients, suggesting that KGF might reflect fibroblast activation after epithelial damage. A randomized controlled trial by McAuley et al. [126] revealed that KGF, which was expected to aid ECM repair, did not improve and even worsened outcomes in ARDS patients, challenging the notion that matrix repair alone can benefit VILI patients. This result underscores the complexities of applying ECM-targeted therapies in clinical settings, highlighting the delicate balance required in ECM management, the multifaceted nature of ARDS and VILI, and the need for precise timing, dosage, and patient selection in treatments.

Such interventions aim to address the underlying causes of lung injury, combining efforts to protect the ECM, reduce inflammation, and enhance tissue repair [127]. Although promising, their clinical application requires further research to optimize their use, considering potential side effects and the balance between therapeutic benefits and risks [127].

The development of advanced imaging techniques and diagnostic tools is poised to revolutionize the real-time assessment of ECM changes and lung tissue damage [128]. Innovations in imaging technologies are expected to enable clinicians to visualize ECM alterations and lung injury with unprecedented detail and in real time [129]. These advancements will likely incorporate machine learning algorithms to enhance image analysis, providing more accurate and timely diagnostics.

Regenerative medicine, particularly through strategies such as stem cell therapy and ECM replacement, holds promise for repairing damaged lung tissue [130]. Ongoing research is focused on developing bioengineered ECM scaffolds that closely mimic the natural lung matrix, providing a conducive environment for tissue regeneration [131]. Progress in this area could lead to breakthrough therapies for conditions previously deemed irreversible [132], transforming the treatment landscape for lung diseases.

The advent of 'Omics' technologies—encompassing genomics, proteomics, metabolomics, and more—has revolutionized our ability to study diseases at a molecular level, offering unprecedented insights into the complex biological processes underpinning health and disease [133]. In the context of investigating the ECM, and specifically the interstitial ECM, these technologies present both unique opportunities and significant challenges [134].

One of the primary opportunities afforded by 'Omics' approaches is the comprehensive profiling of the ECM composition and the dynamic changes it undergoes in response to mechanical ventilation and other stressors [135]. For instance, proteomics can elucidate the intricate array of proteins that constitute the interstitial ECM, identifying alterations in collagen, elastin, fibronectin, and other matrix proteins that are pivotal to lung structure and function [136]. Similarly, metabolomics can reveal changes in the biochemical environment within the ECM that may influence cell–matrix interactions, tissue repair, and fibrosis [136]. Such detailed molecular characterization can uncover new biomarkers of ECM damage and potential therapeutic targets to VILI [137].

However, improving the specificity of 'Omics' technologies in order to distinguish the interstitial ECM from origins in other lung structures presents a significant challenge [138]. The lung's complex architecture, comprising various cell types, blood vessels, and airspaces, intertwined with the ECM, means that 'Omics' analyses often capture a composite snapshot of this heterogeneous environment [138]. This can confound the interpretation of data, making it difficult to attribute observed molecular changes specifically to the interstitial ECM [139].

To address this challenge, investigators can employ targeted approaches within 'Omics' studies to enhance specificity for the interstitial ECM [140]. Advanced sample preparation techniques, such as laser capture microdissection, enable the isolation of specific tissue regions enriched in interstitial ECM for analysis [140, 141]. Additionally, integrating 'Omics' data with spatially resolved techniques, such as imaging mass spectrometry or single-cell RNA sequencing, can provide contextual insights into where specific ECM components are altered within the lung tissue [140]. Such integrative approaches can help delineate the molecular signature of the interstitial ECM from confounding structures, offering a more precise understanding of its role in lung pathology.

The incorporation of computational modeling into the study of lung mechanics and pathology presents a promising avenue to dissect the complex contributions of different factors, including the interstitial ECM and surfactant, to lung function and response to injury [142]. Computational models offer a unique framework for simulating lung physiology and pathology, allowing for the isolation and manipulation of variables in ways that are not feasible in experimental or clinical settings [143].

Specifically, computational models can be designed to simulate the mechanical properties of the lung's interstitial ECM, such as elasticity, stiffness, and resistance to deformation [142]. By adjusting these parameters, researchers can evaluate how changes in the ECM, akin to those induced by mechanical ventilation or disease, influence lung function [142]. This fine tuning includes the ability to simulate the effects of ECM degradation, fibrosis, and altered matrix composition on lung compliance, gas-exchange efficiency, and susceptibility to injury [142].

Moreover, computational modeling can facilitate the exploration of how interventions aimed at preserving or restoring ECM integrity, such as the administration of ECM-protective agents or regenerative therapies, might interact with surfactant therapy in the management of lung diseases [144]. This includes predicting outcomes of combined treatments and identifying optimal therapeutic windows and dosages [144]. The challenge lies in the development of sufficiently detailed and accurate models that faithfully represent the complexities of lung structure and function [142].

To advance our understanding of VILI and the role of ECM, future research should focus on developing and applying techniques capable of distinguishing between interstitial and intraluminal ECM changes [145]. Innovative imaging techniques, molecular markers, and computational models may offer new insights into compartment-specific ECM dynamics [146]. Such research is essential for identifying targeted interventions that can mitigate the adverse effects of mechanical ventilation on lung structure and function.

## Conclusions

The ECM plays an important role in the formation of VILI. The main process is the shedding of GAGs and fragmentation of PGs caused by mechanical stress. Reduced lymphatic drainage may cause increased interstitial volume and promote ECM damage, and the abnormal distribution of pressure may lead to excessive mechanical stress on the ECM. Less GAG shedding occurs in spontaneous breathing than in mechanical ventilation, and mechanical ventilation at higher V_T_s causes more ECM damage than mechanical ventilation at lower V_T_s. GAG shedding and PG fragmentation may lead to the activation of proinflammatory processes, immune responses, and fibrosis. MMP activation due to GAG shedding seems to be the main driving factor of damage to the host because it can degrade several ECM components. Ventilation using PEEP is protective against GAG damage, although overdistention may stimulate lung fibrogenesis.

Moreover, the inability to differentiate between interstitial and intraluminal ECM remodeling presents a notable challenge in translating research findings into clinical practice. This limitation underscores the complexity of ECM dynamics in the lung and suggests that our understanding of VILI’s pathogenesis remains incomplete. Recognizing this gap is crucial for interpreting current research and for guiding future studies aimed at unraveling the distinct roles of ECM compartments in lung injury and repair.

## Data Availability

The data supporting the findings of this study are available within the article.

## Abbreviations

ARDS: Acute respiratory distress syndrome
CC-16: Club cell secretory protein-16
CS-PG: Chondroitin sulfate-proteoglycan
DS-PG: Dermatan sulfate-proteoglycan
ECM: Extracellular matrix
GAGs: Glycosaminoglycans
HS-PG: Heparan sulfate-proteoglycan
IL: Interleukin
JNK: C-Jun N-terminal kinase
KGF: Keratinocyte growth factor
MMPs: Matrix metalloproteinases
PCIII: Type III procollagen
PCV: Pressure-controlled ventilation
PEEP: Positive end-expiratory pressure
Pi: Hydrostatic pressure of the interstitium
RAGE: Receptor for advanced glycation end-products
RMs: Recruitment maneuvers
TGF: Transforming growth factor
TIMPs: Tissue inhibitors of metalloproteinases
VCAM-1: Vascular cell adhesion molecule 1
VILI: Ventilator-induced lung injury
V_T_: Tidal volume
